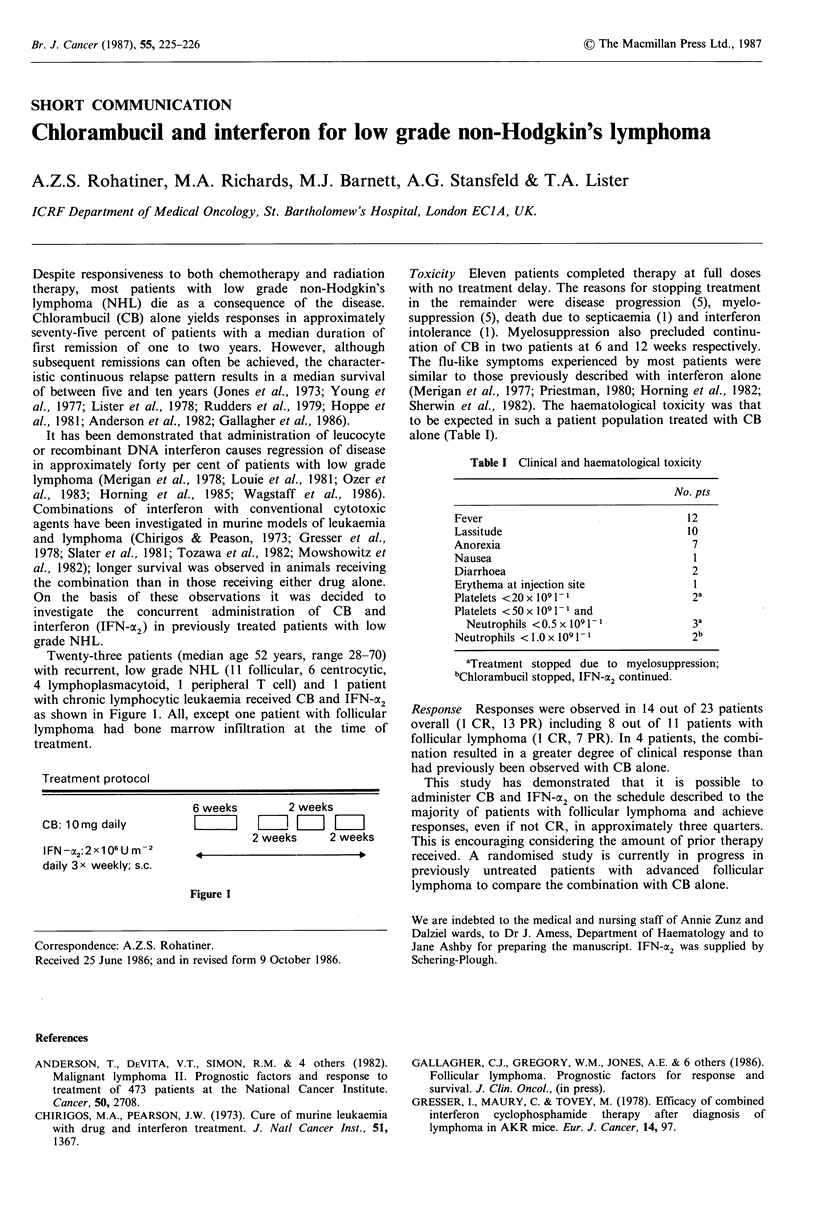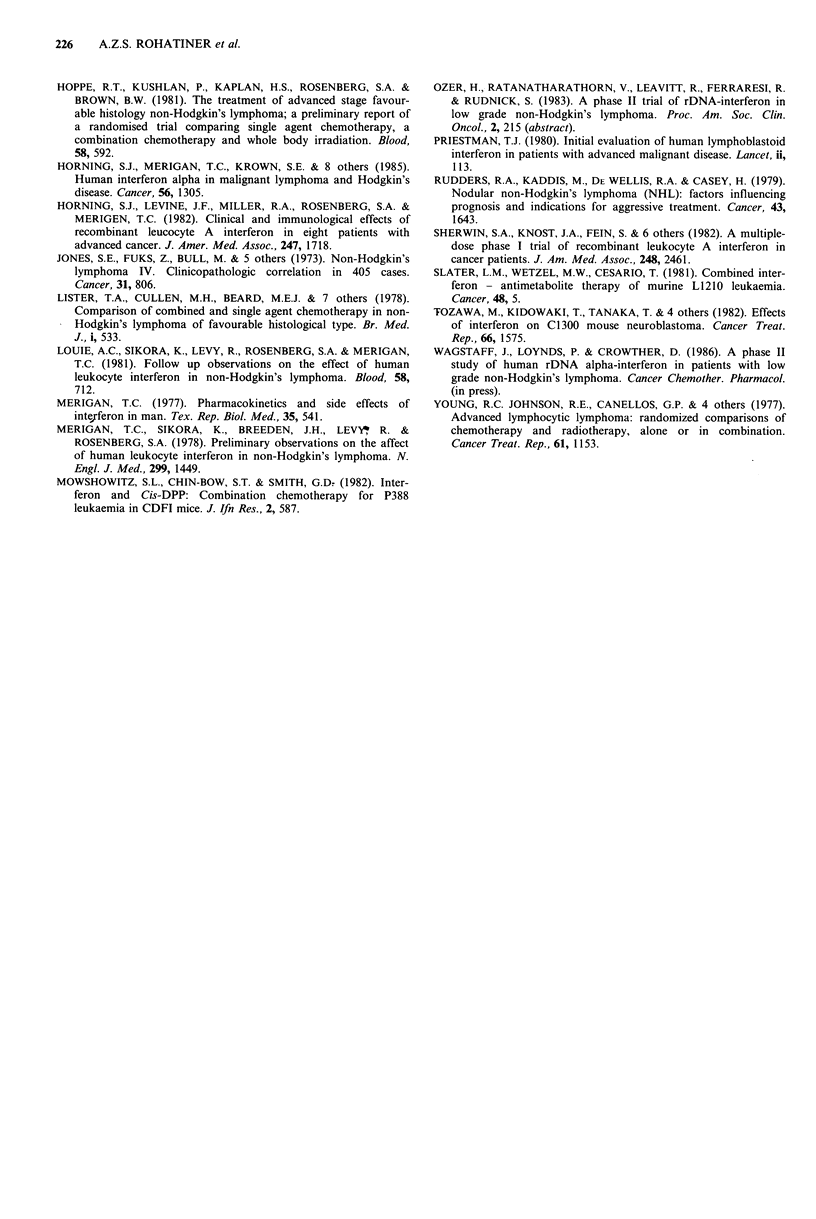# Chlorambucil and interferon for low grade non-Hodgkin's lymphoma.

**DOI:** 10.1038/bjc.1987.42

**Published:** 1987-02

**Authors:** A. Z. Rohatiner, M. A. Richards, M. J. Barnett, A. G. Stansfeld, T. A. Lister


*Br. J. Cancer* (1987), **55**, 225–226 © The Macmillan Press Ltd., 1987

SHORT COMMUNICATION

### Chlorambucil and interferon for low grade non-Hodgkin's lymphoma

A.Z.S. Rohatiner, M.A. Richards, M.J. Barnett, A.G. Stansfeld & T.A. Lister

*ICRF Department of Medical Oncology, St. Bartholomew's Hospital, London EC1A, UK.*

Despite responsiveness to both chemotherapy and radiation therapy, most patients with low grade non-Hodgkin's lymphoma (NHL) die as a consequence of the disease. Chlorambucil (CB) alone yields responses in approximately seventy-five percent of patients with a median duration of first remission of one to two years. However, although subsequent remissions can often be achieved, the characteristic continuous relapse pattern results in a median survival of between five and ten years (Jones *et al.*, 1973; Young *et al.*, 1977; Lister *et al.*, 1978; Rudders *et al.*, 1979; Hoppe *et al.*, 1981; Anderson *et al.*, 1982; Gallagher *et al.*, 1986).

It has been demonstrated that administration of leucocyte or recombinant DNA interferon causes regression of disease in approximately forty per cent of patients with low grade lymphoma (Merigan *et al.*, 1978; Louie *et al.*, 1981; Ozer *et al.*, 1983; Horning *et al.*, 1985; Wagstaff *et al.*, 1986). Combinations of interferon with conventional cytotoxic agents have been investigated in murine models of leukaemia and lymphoma (Chirigos & Peason, 1973; Gresser *et al.*, 1978; Slater *et al.*, 1981; Tozawa *et al.*, 1982; Mowshowitz *et al.*, 1982); longer survival was observed in animals receiving the combination than in those receiving either drug alone. On the basis of these observations it was decided to investigate the concurrent administration of CB and interferon (IFN-$\alpha_2$) in previously treated patients with low grade NHL.

Twenty-three patients (median age 52 years, range 28–70) with recurrent, low grade NHL (11 follicular, 6 centrocytic, 4 lymphoplasmacytoid, 1 peripheral T cell) and 1 patient with chronic lymphocytic leukaemia received CB and IFN-$\alpha_2$ as shown in Figure 1. All, except one patient with follicular lymphoma had bone marrow infiltration at the time of treatment.

**Figure 1**

Correspondence: A.Z.S. Rohatiner.
Received 25 June 1986; and in revised form 9 October 1986.

*Toxicity* Eleven patients completed therapy at full doses with no treatment delay. The reasons for stopping treatment in the remainder were disease progression (5), myelosuppression (5), death due to septicaemia (1) and interferon intolerance (1). Myelosuppression also precluded continuation of CB in two patients at 6 and 12 weeks respectively. The flu-like symptoms experienced by most patients were similar to those previously described with interferon alone (Merigan *et al.*, 1977; Priestman, 1980; Horning *et al.*, 1982; Sherwin *et al.*, 1982). The haematological toxicity was that to be expected in such a patient population treated with CB alone (Table I).

**Table I** Clinical and haematological toxicity

| | *No. pts* |
|---|---|
| Fever | 12 |
| Lassitude | 10 |
| Anorexia | 7 |
| Nausea | 1 |
| Diarrhoea | 2 |
| Erythema at injection site | 1 |
| Platelets $<20\times10^9\,l^{-1}$ | 2[a] |
| Platelets $<50\times10^9\,l^{-1}$ and Neutrophils $<0.5\times10^9\,l^{-1}$ | 3[a] |
| Neutrophils $<1.0\times10^9\,l^{-1}$ | 2[b] |

[a]Treatment stopped due to myelosuppression; [b]Chlorambucil stopped, IFN-$\alpha_2$ continued.

*Response* Responses were observed in 14 out of 23 patients overall (1 CR, 13 PR) including 8 out of 11 patients with follicular lymphoma (1 CR, 7 PR). In 4 patients, the combination resulted in a greater degree of clinical response than had previously been observed with CB alone.

This study has demonstrated that it is possible to administer CB and IFN-$\alpha_2$ on the schedule described to the majority of patients with follicular lymphoma and achieve responses, even if not CR, in approximately three quarters. This is encouraging considering the amount of prior therapy received. A randomised study is currently in progress in previously untreated patients with advanced follicular lymphoma to compare the combination with CB alone.

We are indebted to the medical and nursing staff of Annie Zunz and Dalziel wards, to Dr J. Amess, Department of Haematology and to Jane Ashby for preparing the manuscript. IFN-$\alpha_2$ was supplied by Schering-Plough.

### References

ANDERSON, T., DEVITA, V.T., SIMON, R.M. & 4 others (1982). Malignant lymphoma II. Prognostic factors and response to treatment of 473 patients at the National Cancer Institute. *Cancer*, **50**, 2708.

CHIRIGOS, M.A., PEARSON, J.W. (1973). Cure of murine leukaemia with drug and interferon treatment. *J. Natl Cancer Inst.*, **51**, 1367.

GALLAGHER, C.J., GREGORY, W.M., JONES, A.E. & 6 others (1986). Follicular lymphoma. Prognostic factors for response and survival. *J. Clin. Oncol.*, (in press).

GRESSER, I., MAURY, C. & TOVEY, M. (1978). Efficacy of combined interferon cyclophosphamide therapy after diagnosis of lymphoma in AKR mice. *Eur. J. Cancer*, **14**, 97.

HOPPE, R.T., KUSHLAN, P., KAPLAN, H.S., ROSENBERG, S.A. & BROWN, B.W. (1981). The treatment of advanced stage favourable histology non-Hodgkin's lymphoma; a preliminary report of a randomised trial comparing single agent chemotherapy, a combination chemotherapy and whole body irradiation. *Blood*, **58**, 592.

HORNING, S.J., MERIGAN, T.C., KROWN, S.E. & 8 others (1985). Human interferon alpha in malignant lymphoma and Hodgkin's disease. *Cancer*, **56**, 1305.

HORNING, S.J., LEVINE, J.F., MILLER, R.A., ROSENBERG, S.A. & MERIGEN, T.C. (1982). Clinical and immunological effects of recombinant leucocyte A interferon in eight patients with advanced cancer. *J. Amer. Med. Assoc.*, **247**, 1718.

JONES, S.E., FUKS, Z., BULL, M. & 5 others (1973). Non-Hodgkin's lymphoma IV. Clinicopathologic correlation in 405 cases. *Cancer*, **31**, 806.

LISTER, T.A., CULLEN, M.H., BEARD, M.E.J. & 7 others (1978). Comparison of combined and single agent chemotherapy in non-Hodgkin's lymphoma of favourable histological type. *Br. Med. J.*, **i**, 533.

LOUIE, A.C., SIKORA, K., LEVY, R., ROSENBERG, S.A. & MERIGAN, T.C. (1981). Follow up observations on the effect of human leukocyte interferon in non-Hodgkin's lymphoma. *Blood*, **58**, 712.

MERIGAN, T.C. (1977). Pharmacokinetics and side effects of interferon in man. *Tex. Rep. Biol. Med.*, **35**, 541.

MERIGAN, T.C., SIKORA, K., BREEDEN, J.H., LEVY, R. & ROSENBERG, S.A. (1978). Preliminary observations on the affect of human leukocyte interferon in non-Hodgkin's lymphoma. *N. Engl. J. Med.*, **299**, 1449.

MOWSHOWITZ, S.L., CHIN-BOW, S.T. & SMITH, G.D. (1982). Interferon and *Cis*-DPP: Combination chemotherapy for P388 leukaemia in CDFI mice. *J. Ifn Res.*, **2**, 587.

OZER, H., RATANATHARATHORN, V., LEAVITT, R., FERRARESI, R. & RUDNICK, S. (1983). A phase II trial of rDNA-interferon in low grade non-Hodgkin's lymphoma. *Proc. Am. Soc. Clin. Oncol.*, **2**, 215 (*abstract*).

PRIESTMAN, T.J. (1980). Initial evaluation of human lymphoblastoid interferon in patients with advanced malignant disease. *Lancet*, **ii**, 113.

RUDDERS, R.A., KADDIS, M., DE WELLIS, R.A. & CASEY, H. (1979). Nodular non-Hodgkin's lymphoma (NHL): factors influencing prognosis and indications for aggressive treatment. *Cancer*, **43**, 1643.

SHERWIN, S.A., KNOST, J.A., FEIN, S. & 6 others (1982). A multiple-dose phase I trial of recombinant leukocyte A interferon in cancer patients. *J. Am. Med. Assoc.*, **248**, 2461.

SLATER, L.M., WETZEL, M.W., CESARIO, T. (1981). Combined interferon – antimetabolite therapy of murine L1210 leukaemia. *Cancer*, **48**, 5.

TOZAWA, M., KIDOWAKI, T., TANAKA, T. & 4 others (1982). Effects of interferon on C1300 mouse neuroblastoma. *Cancer Treat. Rep.*, **66**, 1575.

WAGSTAFF, J., LOYNDS, P. & CROWTHER, D. (1986). A phase II study of human rDNA alpha-interferon in patients with low grade non-Hodgkin's lymphoma. *Cancer Chemother. Pharmacol.* (in press).

YOUNG, R.C. JOHNSON, R.E., CANELLOS, G.P. & 4 others (1977). Advanced lymphocytic lymphoma: randomized comparisons of chemotherapy and radiotherapy, alone or in combination. *Cancer Treat. Rep.*, **61**, 1153.